# Wire-guided cannulation over a pancreatic stent versus double guidewire technique in patients with difficult biliary cannulation

**DOI:** 10.1186/s12876-015-0381-4

**Published:** 2015-10-28

**Authors:** Min Jae Yang, Jae Chul Hwang, Byung Moo Yoo, Jin Hong Kim, Hyoung-Kyu Ryu, Soon Sun Kim, Joon Koo Kang, Min Kyeong Kim

**Affiliations:** Department of Gastroenterology, Ajou University School of Medicine, San-5, Woncheon-dong, Yongtong-gu, 443-721, Suwon South Korea; Medical Information and Media Center, Ajou University School of Medicine, San-5, Woncheon-dong, Yongtong-gu, 443-721, Suwon South Korea

**Keywords:** Endoscopic retrograde cholangiopancreatography, Cannulation, Pancreatitis, Bile duct, Pancreatic duct

## Abstract

**Background:**

In cases of difficult bile duct cannulation, the use of wire-guided cannulation over a pancreatic stent (WGC-PS) or the double guidewire technique (DGT) may facilitate biliary cannulation. The aim of this study was to compare the outcomes of WGC-PS and DGT in patients with difficult biliary cannulation.

**Methods:**

We conducted a retrospective cohort study of all endoscopic retrograde cholangiopancreatographies (ERCPs) performed between July 2009 and November 2014 at a single tertiary referral center. WGC-PS or DGT was performed in patients for whom biliary cannulation was difficult and guidewire insertion into the pancreatic duct (PD) was inadvertently achieved while attempting the standard WGC technique. In those cases, we used the WGC-PS technique from July 2009 to January 2012 (WGC-PS group), and the DGT technique from February 2012 to November 2014 (DGT group). In the DGT group, WGC-PS was sequentially performed if successful biliary cannulation was not achieved during the DGT attempt. Consecutive patients who underwent DGT and/or WGC-PS with the aim of selective biliary cannulation were enrolled. The primary outcome parameter was the rate of initial successful biliary cannulation.

**Results:**

During the study period 3270 ERCPs were performed and a total of 177 patients were enrolled. The rate of initial successful cannulation was 66.7 % (60/90) in the WGC-PS group and 70.1 % (61/87) in the DGT group (*P* = 0.632). In 26 cases of failed DGT, WGC-PS was sequentially performed in the DGT group, and cannulation was successful in 14 of these patients. The rate of successful cannulation without the needle-knife precut technique was significantly higher in the DGT group compared with the WGC-PS group (75/87, 86.2 % vs. 60/90, 66.7 %, *P* = 0.003). The incidence of post-ERCP pancreatitis was 3.3 % (3/90) in the WGC-PS group and 10.3 % (9/87) in the DGT group (*P* = 0.077).

**Conclusions:**

In patients for whom biliary cannulation was difficult and PD access was inadvertently achieved while attempting the standard WGC technique, both WGC-PS and DGT were equally effective. Furthermore, the stepwise approach using DGT followed by WGC-PS as needed facilitated successful biliary cannulation and reduced the need for the needle-knife precut technique.

## Background

Selective biliary cannulation is essential for the success of therapeutic endoscopic biliary intervention. The success rate for biliary cannulation with conventional methods ranges between 50 and 90 %, depending on the experience of the endoscopist, patient anatomy and disease factors [1–5]. In some cases, bile duct cannulation can be difficult because of special anatomical features, inflammatory processes, or adenomas of the papilla or periampullary diverticulum [6]. Prolonged papillary manipulation for cannulation of the bile duct in these patients increases the risk of endoscopic retrograde cholangiopancreatography (ERCP)-related complications [7, 8]. Various techniques, such as the double guidewire technique (DGT), wire-guided cannulation over a pancreatic stent (WGC-PS), the needle-knife precut technique, and transpancreatic sphincterotomy, have been used to improve the success rate of biliary cannulation [6, 9–11].

One method to facilitate biliary cannulation is DGT, which involves preinserting a guidewire into the pancreatic duct (PD). Since its first description by Dumonceau et al. [12], DGT has been performed with promising results in cases of difficult biliary cannulation, particularly in patients with a papilla that is prominent with a tortuous intraduodenal segment or located in a duodenal diverticulum [12, 13].

WGC-PS, which is another technique for difficult biliary cannulation, was first described by Coté et al. [11]. In cases in which attempts at biliary cannulation result in repeated PD cannulation, the placement of a plastic stent protects the pancreatic orifice and facilitates biliary cannulation [11]. The use of a PD stent is an attractive option because multiple cannulation attempts are an independent risk factor for post-ERCP pancreatitis (PEP) [14]. Additionally, PD stents may decrease the risk and severity of PEP in high-risk situations, such as difficult cannulation or during the needle-knife precut technique [15, 16]. A limited amount of available data supports the efficacy and safety of this technique. Therefore, the aim of this study was to compare the outcomes between WGC-PS and DGT in patients with difficult biliary cannulation.

## Methods

### Patients and data

We conducted a retrospective cohort study of all ERCPs performed between July 2009 and November 2014 at a single tertiary referral hospital (Ajou University Hospital, Suwon, Korea). During the study period, WGC-PS or DGT was performed in patients for whom biliary cannulation was difficult and guidewire insertion into the PD was achieved by unintentional PD cannulation while attempting the standard WGC technique. In those patients, we used the WGC-PS technique between July 2009 and January 2012 (WGC-PS group), while the DGT technique was performed from February 2012 to November 2014 (DGT group). In the WGC-PS group, needle-knife fistulotomy (NKF) was performed if successful biliary cannulation was not achieved within 5 min during the WGC-PS attempt. In the DGT group, WGC-PS was sequentially performed if successful biliary cannulation was not achieved within 5 min while attempting DGT, and NKF was performed if successful biliary cannulation was not achieved within an additional 5 min during the WGC-PS attempt. DGT could be attempted before WGC-PS for biliary cannulation; we believed that the stepwise approach using DGT followed by WGC-PS as needed could facilitate successful biliary cannulation. Thus, we have performed the stepwise approach since February 2012 in patients for whom biliary cannulation was difficult and guidewire insertion into the PD was achieved by unintentional PD cannulation while attempting the standard WGC technique. Consecutive patients who underwent DGT and/or WGC-PS with the aim of selective biliary cannulation were included. Exclusion criteria were (1) previous sphincterotomy or endoscopic papillary balloon dilation, (2) ERCP for pancreatic intervention, (3) Billoth II or Roux-en-Y anatomy, (4) acute pancreatitis, (5) successful selective biliary cannulation with a standard cannulation technique, and (6) the use of the needle-knife precut technique immediately after initial failure of the standard cannulation technique. We performed the needle-knife precut technique immediately after initial failure of the standard cannulation technique in patients for whom biliary cannulation was difficult and unintentional PD cannulation with a guidewire was not achieved while attempting the standard WGC technique. ERCP data were prospectively collected in our ERCP database at Ajou University Hospital. The database included patient characteristics (e.g., age, sex, diagnosis, history of surgery, and presence of periampullary diverticulum), procedure data (e.g., type of endoscope, premedication, cannulation method, cannulation success, and ERCP maneuvers), and procedure-related complications. Informed consent was obtained from all patients before the procedure, and this study was approved by the Institutional Review Board of Ajou University Hospital (AJIRB-MED-MDB-15-094).

### Definitions and outcome parameters

Difficult biliary cannulation was defined as unsuccessful cannulation within 10 min of attempting the conventional method. Initial success of cannulation was defined as the achievement of deep biliary cannulation at the time of the first attempted WGC-PS or DGT. ERCP-related complications were defined and graded according to the 1991 consensus guidelines [17]. PEP was defined as new or worsened abdominal pain and elevated serum amylase levels exceeding the upper limit of normal by at least three-fold within 24 h of the procedure. Clinically significant bleeding was defined as the presence of melena, hematochezia, or hematemesis associated with a decrease in hemoglobin of at least 2 g/dl or the need for blood transfusion. Perforation included retroperitoneal or bowel wall perforation that was documented by radiographic images. The primary outcome parameter was the rate of initial successful biliary cannulation. The secondary outcome measures were the rate of successful biliary cannulation without the needle-knife precut technique, the rate of overall successful biliary cannulation, and the rate of PEP.

### Endoscopic procedures

ERCP was performed with side-viewing endoscopes (JF-240, JF-260 V, and TJF-260 V; Olympus Optical Co., Ltd, Tokyo, Japan) under sedation with a standard dose of midazolam, propofol, and meperidine. Selective biliary cannulation was first attempted by the standard WGC technique with a 0.035-in. guidewire (Jagwire; Boston Scientific, Natick, MA, USA or Tracer Metro® Direct™ Wire Guide; Wilson-Cook Medical Inc., Winston-Salem, NC, USA or Fusion® LoopTip™ Wire Guide; Wilson-Cook Medical Inc., Winston-Salem, NC, USA). A second endoscopist who was experienced in assisting with ERCP procedures manipulated the guidewire during WGC.

**Fig. 1:**
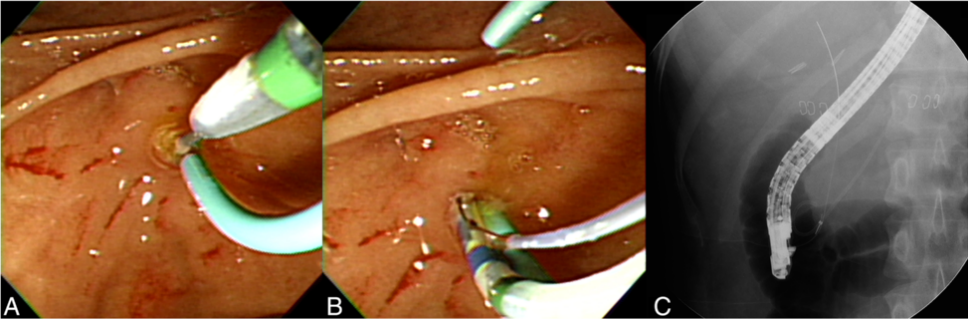
Wire-guided cannulation over a pancreatic stent for biliary cannulation. **a** After a 5 F pancreatic duct stent was placed, cannulation of the bile duct was attempted using a sphincterotome, which was directed to the biliary orifice at its usual 10–11 o’clock position in relation to the pancreatic duct stent. **b**, **c** Successful biliary cannulation with a guidewire was achieved after the previous insertion of the 5 F pancreatic duct stent

WGC-PS was performed as follows. After a guidewire was placed into the PD, a polyethylene stent (5 F and 5-cm, Zimmon stent, Cook Ireland Ltd., Limerick, Ireland) was placed into the main PD. Cannulation of the bile duct then was attempted using a sphincterotome, which was preloaded with a guidewire. The sphincterotome was directed to the biliary orifice at its usual 10–11 o’clock position on the ampulla using the PD stent as a reference point. WGC of the bile duct was attempted over the PD stent (Fig. 1).

**Fig. 2:**
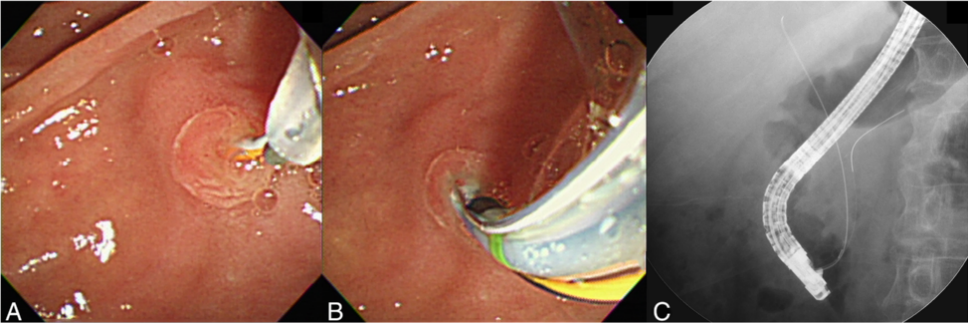
Double guidewire technique for biliary cannulation. **a** Bile duct cannulation was aimed upward to the 10–11 o’clock position in relation to the pancreatic wire. **b**, **c** Successful biliary cannulation with a second guidewire was achieved after the previous insertion of the first guidewire into the pancreatic duct

DGT was performed as follows. A guidewire was inserted into the PD to at least half of the presumed total length of the PD (guided by fluoroscopy). A sphincterotome was reinserted along the first guidewire after being reloaded with the second guidewire. The tip of the device was positioned in the ampulla, bending over the pancreatic wire and targeting the 10–11 o’clock position on the ampullary orifice, to attempt cannulation of the bile duct. WGC of the bile duct was attempted alongside the pancreatic wire (Fig. 2). After successful biliary cannulation, the pancreatic wire was removed from the PD with or without pancreatic stenting at the discretion of the endoscopist.

NKF was performed as follows. An incision with a needle knife that was preloaded with a guidewire was made near the junction of the upper third and the lower two thirds of the ampullary mound, creating a direct bilio-enteral fistula to gain access. The contrast agent was not injected until deep biliary cannulation with a guidewire had been achieved. All endoscopic procedures were performed by three endoscopists, each of whom performs more than 200 ERCP procedures per year.

### Statistical analysis

Analyses were performed using SPSS 14.0 (SPSS Inc., Chicago, IL, USA). Continuous variables were compared with Student’s *t* test. Categorical variables were tested by the chi-square test or Fisher’s exact test. Statistical significance was set at a *P* value of < 0.05.

## Results

A total of 3270 ERCPs, including 2528 patients with a native papilla, were performed during the study period. Of the 2528 patients with a native papilla, we excluded 52 ERCPs performed for pancreatic intervention, 50 patients with surgically altered gastrointestinal anatomy, and 202 patients with comorbid acute pancreatitis. Among the remaining 2224 patients, we excluded 1871 ERCPs in which selective biliary cannulation was achieved with a standard cannulation technique and 176 ERCPs in which the needle-knife precut technique was attempted immediately after initial failure of the standard cannulation technique. Finally, a total of 177 patients who underwent DGT and/or WGC-PS were enrolled in this study. The characteristics of the patients are summarized in Table 1. There were no significant differences with respect to sex, age, periampullary diverticulum, cholecystectomy status, and ERCP indication between the two groups. The only significant difference was a higher percentage of PD stent placement in the WGC-PS group compared with the DGT group (100 % vs. 56.3 %, *P* < 0.001).

**Table 1:** Baseline characteristics

	WGC-PS (*n* = 90)	DGT (*n* = 87)	*P* value
Sex (male/female)	49/41	44/43	0.653
Age (mean ± SD)	54.2 ± 16.4	57.3 ± 16.7	0.221
Periampullary diverticulum, n (%)	32 (35.6)	33 (37.9)	0.758
Cholecystectomy, n (%)	11 (12.2)	11 (12.6)	1.000
Indication for ERCP			0.174
CBD stone, n (%)	69 (76.7)	71 (81.6)	
Malignant biliary stricture, n (%)	15 (16.7)	16 (18.4)	
Benign biliary stricture, n (%)	3 (3.3)	0	
Bile leak, n (%)	3 (3.3)	0	
Pancreatic duct stent, n (%)	90 (100)	49 (56.3)	<0.001

*WGC-PS* wire-guided cannulation over a pancreatic stent, *DGT* double guidewire technique, *SD* standard deviation, *ERCP* endoscopic retrograde cholangiopancreatography, *CBD* common bile duct

Of the 90 patients in the WGC-PS group, initial successful biliary cannulation was achieved in 60 (66.7 %) patients. NKF was performed in the 30 patients with failed biliary cannulation, and 27 patients had successful biliary cannulation. In one patient, ERCP was re-attempted, and successful biliary cannulation was achieved with NKF. Three patients underwent percutaneous transhepatic biliary drainage following failed biliary cannulation with NKF. The overall success rate of biliary cannulation was 96.6 % (87/90) in the WGC-PS group.

Of the 87 patients in the DGT group, initial successful biliary cannulation was achieved in 61 (70.1 %) patients. In 26 patients with a failed DGT, WGC-PS was sequentially performed, and biliary cannulation was successful in 14 of these patients. One patient underwent a second ERCP, and successful biliary cannulation was achieved with WGC-PS. NKF was performed in the 12 patients who had failed biliary cannulation with WGC-PS, and 11 patients had successful biliary cannulation. One patient underwent percutaneous transhepatic biliary drainage following failed biliary cannulation with NKF. The overall success rate of biliary cannulation was 98.9 % (86/87) in the DGT group.

There were no significant differences in the initial and overall successful biliary cannulation rates between the two groups (Table 2). The rate of successful biliary cannulation without NKF was significantly higher in the DGT group than in the WGC-PS group (86.2 % vs.66.7 %, *P* = 0.003).

**Table 2:** Outcomes of the WGC-PS and DGT groups

	WGC-PS (*n* = 90)	DGT (*n* = 87)	*P* value
Initial success rate, n (%)	60 (66.7)	61 (70.1)	0.632
Success rate without NKF, n (%)	60 (66.7)	75 (86.2)^*^	0.003
Overall success rate, n (%)	87 (96.6)	86 (98.9)	1.000
Pancreatitis, total, n (%)	3 (3.3)	9 (10.3)	0.077
Mild, n	3	7	
Moderate, n	0	2	
Bleeding, n	0	0	
Perforation, n	0	0	

*WGC-PS* wire-guided cannulation over a pancreatic stent, *DGT* double guidewire technique, *NKF* needle-knife fistulotomy

^*^In 26 patients who had failed DGT, WGC-PS was sequentially performed, and cannulation was successful in 14 of these patients

Post-ERCP complications are summarized in Table 2. PEP developed in 3.3 % (three patients, mild) of patients in the WGC-PS group and 10.3 % (seven patients, mild and two patients, moderate) of patients in the DGT group (*P* = 0.077). In the patients with PEP in the WGC-PS group, successful biliary cannulation was achieved with WGC-PS in two patients and with NKF in one patient. In the patients with PEP in the DGT group, successful biliary cannulation was achieved with DGT in eight patients and with WGC-PS in one patient. Among them, placement of a PD stent was performed in two patients, and these cases of pancreatitis were mild. The rate of PEP was not significantly different between the patients with pancreatic stenting in the DGT group and the patients in the WGC-PS group (2/49, 4.1 % vs. 3/90, 3.3 %, *P* = 1.000). The rate of PEP was significantly higher in the patients without pancreatic stenting in the DGT group than the patients in the WGC-PS group (7/38, 18.4 % vs. 3/90, 3.3 %, *P* = 0.007). In the DGT group, the rate of PEP was significantly higher in the patients without pancreatic stenting than the patients with pancreatic stenting (7/38, 18.4 % vs. 2/49, 4.1 %, *P* = 0.037). No bleeding or perforation occurred in either group.

## Discussion

Various techniques have been developed for selective deep biliary cannulation, which requires skill and experience. The needle-knife precut technique is frequently used to overcome failed standard biliary cannulation and is highly successful when performed by an expert endoscopist. However, a disadvantage of this method is its higher rate of complications (6 to >20 %), including bleeding, perforation, and pancreatitis [18–20]. Some studies have suggested increased numbers of complications with the needle-knife precut technique when attempted by physicians who perform these procedures less than once per week [18, 21, 22]. As a less invasive technique, PD guidewire or stent placement has been reported to be effective in patients with difficult biliary cannulation [6, 9, 11, 23–26].

The placement of a PD stent to guide an ultra-tapered cannula for biliary cannulation without the needle-knife precut technique was initially described by Slivka [23]. A retrospective study demonstrated that the use of a standard sphincterotome over a PD stent could facilitate biliary cannulation without requiring the needle-knife precut technique in 41 % (16/39) of patients and reported two (5.1 %) cases of mild PEP [25]. Another retrospective cohort study showed that WGC-PS could facilitate biliary cannulation without the need for the needle-knife precut technique in 78.9 % (60/76) of patients. In that study, there were four (5.3 %) cases of mild PEP [11]. The initial success rate of WGC-PS in our study (66.7 %) was lower than that reported by Coté et al. [11] (78.9 %) but higher than that reported by Goldberg et al. [25] (41 %). There are two explanations that may contribute to this difference. First, in the former study [11], physician-controlled WGC of the bile duct with a short guidewire (260 cm) was attempted over the PD stent in the cases in which PD stents were placed to facilitate biliary cannulation. In our study, the guidewire was controlled by an assistant, and the assistant informed the operator of resistance to the advancement of the guidewire while attempting WGC-PS for biliary cannulation. Although there are no prospective randomized studies comparing physician-controlled WGC with assistant-controlled WGC, the operator should be able to detect the degree of resistance to the advancement of the guidewire while performing physician-controlled WGC. Second, in the latter study [25], biliary cannulation was attempted using a standard sphincterotome without a guidewire over a PD stent in the cases in which PD stents were placed to facilitate biliary cannulation. The placement of the sphincterotome alongside the PD stent within a common channel can be technically demanding, especially in the setting of a small papillary orifice.

In DGT, placement of a pancreatic guidewire has several advantages for selective biliary cannulation, including opening a stenotic papillary orifice, stabilizing the papilla, lifting it toward the working channel, straightening the PD and the common channel, draining the PD, potentially minimizing repeated injections into the PD, and providing access for the placement of a PD stent if necessary [19]. The initial success rate of DGT in our study (70.1 %) is higher than that reported by Herreros de Tejada et al. [27] (47 %) and similar to the rates reported by Ito et al. [26] (73 %) and Angsuwatcharakon et al. [9] (73.9 %). In our study, we used DGT in cases in which biliary cannulation was difficult and guidewire insertion into the PD was inadvertently achieved. In contrast to the present study, in Herreros de Tejada et al.’s prospective randomized study, a significant percentage of failures in the DGT group consisted of cases in which a guidewire could not be inserted in the PD at least once (19/76, 25 %) [27]. Consistent with our study, in Ito et al.’s retrospective study, DGT was performed in 113 patients in whom cannulation of the bile duct was unsuccessful with standard cannulation techniques and guidewire insertion into the PD was achieved [26]. Additionally, in Angsuwatcharakon et al.’s prospective randomized study, 20/23 patients from the DGT group had inadvertent PD cannulation while attempting the standard cannulation technique [9].

**Fig. 3:**
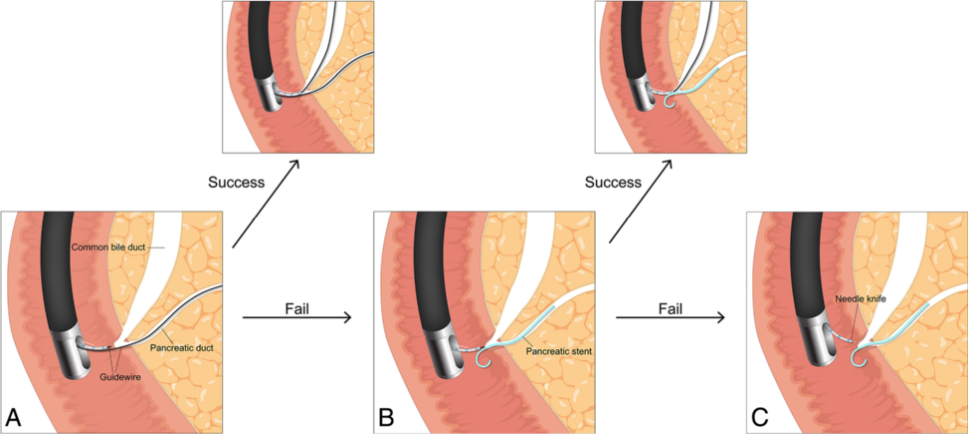
Schematic presentation of the stepwise approach using double guidewire technique followed by wire-guided cannulation over a pancreatic stent as needed. **a** Double guidewire technique. **b** Wire-guided cannulation over a pancreatic stent. **c** Needle knife fistulotomy over a pancreatic stent

In WGC-PS, the placement of a PD stent often can facilitate biliary cannulation by providing clues regarding the optimal angle to further approach to the papilla to obtain biliary access and by blocking the PD orifice from further attempts at cannulation, thus minimizing further pancreatic manipulation [11]. Another potential advantage of WGC-PS is that placement of a PD stent reduces the rate of PEP in patients at high risk for this complication [15, 16, 28]. WGC-PS could be attempted before the needle-knife precut technique in patients in whom DGT failed for biliary cannulation. However, attempting DGT in patients for whom WGC-PS failed would be impractical because this task would require removing the PD stent and advancing a guidewire into the PD a second time. In our study, there was no significant difference in the rates of initial successful biliary cannulation or complications between the two groups. Moreover, in the DGT group, WGC-PS was sequentially performed in 26 patients with a failed DGT, and cannulation was successful in 14 of these patients. The rate of successful biliary cannulation without NKF was significantly higher in the DGT group than in the WGC-PS group. Therefore, we suggest that a stepwise approach using DGT followed by WGC-PS as needed when the standard biliary cannulation technique fails and placement of a PD guidewire is inadvertently achieved is effective and has an acceptable complication profile (Fig. 3).

In our study, the rate of pancreatic stenting was significantly higher in the WGC-PS group than in the DGT group (100 % vs. 56.3 %, *P* < 0.001). Although there was no significant difference in the rate of PEP between the two groups, there was a trend toward a higher rate of PEP in the DGT group compared with the WGC-PS group (10.3 % vs. 3.3 %, *P* = 0.077). In the WGC-PS group, mild PEP occurred in three patients. Of the nine patients with PEP in the DGT group, pancreatic stenting was performed in two patients, and moderate pancreatitis occurred in two patients without pancreatic stenting. The rate of PEP was not significantly different between the patients with pancreatic stenting in the DGT group and the patients in the WGC-PS group (2/49, 4.1 % vs. 3/90, 3.3 %, *P* = 1.000). However, the rate of PEP was significantly higher in the patients without pancreatic stenting in the DGT group than the patients in the WGC-PS group (7/38, 18.4 % vs. 3/90, 3.3 %, *P* = 0.007). Additionally, in the DGT group, the rate of PEP was significantly higher in the patients without pancreatic stenting than the patients with pancreatic stenting (7/38, 18.4 % vs. 2/49, 4.1 %, *P* = 0.037). Maneuvers while attempting DGT, such as rotating or pushing the duodenoscope and adjusting the elevator of the scope for a prolonged period of time when the first guidewire remains in the PD, may damage the papilla and the PD [29]. Yoo et al. [6] used no prophylactic pancreatic stenting in 34 patients in the DGT group, while another study used prophylactic pancreatic stenting in only 12 of 97 patients who were randomized to the DGT group [27], which may explain their high incidences of PEP (38.2 and 17 %, respectively). Therefore, after placement of a PD guidewire for biliary cannulation, prophylactic PD stenting should be considered to reduce the incidence and severity of PEP.

Our study was limited by its retrospective cohort design. We attempted to overcome this limitation by using a prospectively collected ERCP database of consecutive patients during the study period. This study was not a head-to-head comparison of two techniques, and we divided the patients chronologically into two groups, according to the time period during which the techniques for biliary cannulation were used. Therefore, bias-related improvements in the techniques of the operators may have been introduced. Another limitation of the study is that it involved a single center, and the procedures were performed by three experienced endoscopists. Thus, further large-scale prospective multicenter studies are needed to confirm that these results are generally applicable to other centers.

## Conclusions

In cases in which biliary cannulation was difficult and PD access was inadvertently achieved while attempting the standard WGC technique, both WGC-PS and DGT were equally effective. In addition, a stepwise approach using DGT followed by WGC-PS as needed facilitated successful biliary cannulation and reduced the need for the needle-knife precut technique.

## Abbreviations

ERCP: Endoscopic retrograde cholangiopancreatography
DGT: Double guidewire technique
WGC-PS: Wire-guided cannulation over a pancreatic stent
PD: Pancreatic duct
PEP: Post-ERCP pancreatitis
NKF: Needle-knife fistulotomy
